# Spontaneous mutations of the *Zpld1* gene in mice cause semicircular canal dysfunction but do not impair gravity receptor or hearing functions

**DOI:** 10.1038/s41598-019-48835-5

**Published:** 2019-08-27

**Authors:** Sarath Vijayakumar, Sherri M. Jones, Timothy A. Jones, Cong Tian, Kenneth R. Johnson

**Affiliations:** 1 Department of Special Education and Communication Disorders, University of Nebraska, Lincoln, NE USA; 2 The Jackson Laboratory, Bar Harbor, ME USA; 3 Present Address: Biomedical Sciences, Creighton University School of Medicine, Omaha, NE USA

**Keywords:** Mechanisms of disease, Gene expression, Inner ear

## Abstract

The cupula is a gelatinous membrane overlying the crista ampullaris of the semicircular canal, important for sensing rotation of the head and critical for normal balance. Recently the zona pellucida like domain containing 1 protein (ZPLD1, also known as cupulin) was identified in the cupula of fish. Here, we describe two new spontaneous mutations in the mouse *Zpld1* gene, which were discovered by the circling behavior of mutant mice, an indicator of balance dysfunction. The *Zpld1* mutant mice exhibited normal hearing function as assessed by auditory brainstem response (ABR) measurements, and their otolithic organs appeared normal. In the inner ear, *Zpld1* mRNA expression was detected only in the hair cells and supporting cells of the crista ampullaris. Normal vestibular sensory evoked potential (VsEP) responses and abnormal vestibulo-ocular reflex (VOR) responses demonstrated that the vestibular dysfunction of the *Zpld1* mutant mice is caused by loss of sensory input for rotary head movements (detected by cristae ampullaris) and not by loss of input for linear head translations (detected by maculae of the utricle and saccule). Taken together, these results are consistent with ZPLD1 being an important functional component of the cupula, but not tectorial or otoconial membranes.

## Introduction

Dizziness and balance dysfunction adversely affects quality of life and is a prevalent health problem in human populations, and vestibular vertigo related to the inner ear accounts for a considerable percentage of this burden^1^. More than 1 in 20 children in the United States between the ages of 3 and 17 (about 3.3 million) have a dizziness or balance problem, according to survey results from a large (n = 10,954) nationally representative epidemiological study^2^. Evidence from family studies suggests that many cases of vestibular dysfunction are heritable; however, in contrast to the major advancements that have been made in the identification of genes that underlie hearing impairment^3^, genetic contributions to vestibular dysfunction are still largely unknown, probably because of clinical variability and difficulties of diagnosis^4,5^. Benign recurrent vertigo, bilateral vestibulopathy, and Meniere disease are familial vestibular disorders, but confirmed genetic causes for these conditions have not been identified^6,7^. Recently, however, studies using whole exome sequencing have identified several candidate genes for familial Meniere disease including *FAM136A*, *DTNA*, *PRKCB*, *DTP* and *SEMA3D*^8–10^. Although several causative genes have been identified for peripheral vestibular dysfunctions that accompany hearing loss disorders, no genetic causes for nonsyndromic vestibulopathies have yet been identified in humans^4,5,7^. Without the aid of other associated anomalies such as hearing loss, the genetic analysis of vestibular dysfunction in humans is problematic because of the difficulty in achieving reliable diagnoses.

Animal models, which can circumvent some of the problems encountered in human studies, have greatly enhanced our knowledge of the physiology and disease pathologies of the vestibular sensory system^11^. Laboratory mice have proved particularly valuable for analyzing the genetic and molecular bases of peripheral vestibular dysfunction^12^. Vestibular dysfunction in mice can often, but not always, be detected by circling, head tossing, or head tilting behaviors; however, definitive proof requires more direct histological and physiological tests. Investigations of the vestibular system in mutant mice have revealed defects in otoconia, stereocilia and hair cells, hair cell synapses, and ionic homeostasis^12^. Many mutations, such as those causing defects of hair cell function or endolymph homeostasis, affect the entire inner ear and result in both auditory and vestibular dysfunction. Some mutations, however, affect only vestibular function. For example, mice with mutations causing otoconial deficits and loss of gravity receptor function, such as *Nox3*^*het*^, *Noxo1*^*hslt*^, and *Otp1*^*tlt*^, exhibit head tilting behavior but have normal hearing, and *Otx1*^*jv*^ mutant mice, which fail to form a lateral semicircular canal, exhibit circling behavior but have normal gravity receptor function and normal hearing^13^. Mice with a null mutation of *Kcna10*, a potassium channel gene expressed in hair cells of the inner ear, have significant vestibular dysfunction but only a mild elevation in hearing thresholds^14^.

Here we report on the discovery and analyses of two new spontaneous mouse mutations that cause hyperactive circling behavior but do not affect hearing. The mutations, named circler with hearing (*cwh*) and spiral (*sprl*), were shown by genetic mapping and DNA sequence analysis to be different mutations of the zona pellucida like domain containing 1 gene (*Zpld1*). The zona pellucida (ZP) domain is a conserved structural element of about 260 amino acids that has been shown to mediate the polymerization of proteins into fibrils or extracellular matrices^15,16^. The mammalian inner ear has three distinct extracellular matrices that overlie sensory epithelia of the mechanoreceptor organs: the tectorial membrane overlying the organ of Corti in the cochlea, the otoconial membranes overlying the utricular and saccular maculae, and the cupula overlying the crista ampullaris of the semicircular canals. These membranes form intimate contacts with the underlying stereocilia of the sensory hair cells and are critical for detection of sound, gravity, and head movements.

Alpha-tectorin and beta-tectorin are ZP domain glycoproteins found in the otoconial and tectorial membranes^17^, and mutations of the gene for alpha-tectorin cause deafness in humans and mice^18^. Although no ZP domain protein has been reported in the mammalian cupula, the zona pellucida-like domain 1 protein (ZPLD1) was shown to be a major component of the cupula in salmon and given the name cupulin^19^, a term first used to designate a hypothetical protein predicted to be a component of the cupula in mice^17^. ZPLD1 is evolutionarily conserved, with a 73% amino acid identity between salmon and mouse. We sought to test whether ZPLD1 is indeed a component of the mouse cupula and whether it is present in other acellular membranes of the inner ear. We also sought to directly measure vestibular function in *Zpld1* mutant and control mice by vestibular sensory evoked potential (VsEP) and vestibulo-ocular reflex (VOR) measurements. Our findings that the spontaneous *sprl* and *cwh* mutations of the *Zpld1* gene cause vestibular but not auditory dysfunction, and that the vestibular dysfunction is limited to loss of sensory input for rotary head movements and not linear accelerations, are consistent with ZPLD1 being a component of the cupula in mice, without apparent importance to tectorial or otoconial membrane function.

## Results

### Phenotypic effects of the *cwh* and *sprl* mutations

Two new recessive mutations that occurred spontaneously in colonies of mice at The Jackson Laboratory were first identified by their hyperactivity and abnormal circling behavior (Supplementary Videos 1 and 2), which often are indicators of inner ear vestibular dysfunction. The mutant mice appeared to have some hearing ability because they responded to sudden loud noises with a Preyer reflex. On the basis of these behavioral and auditory phenotypes, the independently occurring mutations were named “spiral” (*sprl*) and “circler with hearing” (*cwh*).

**Figure 1:**
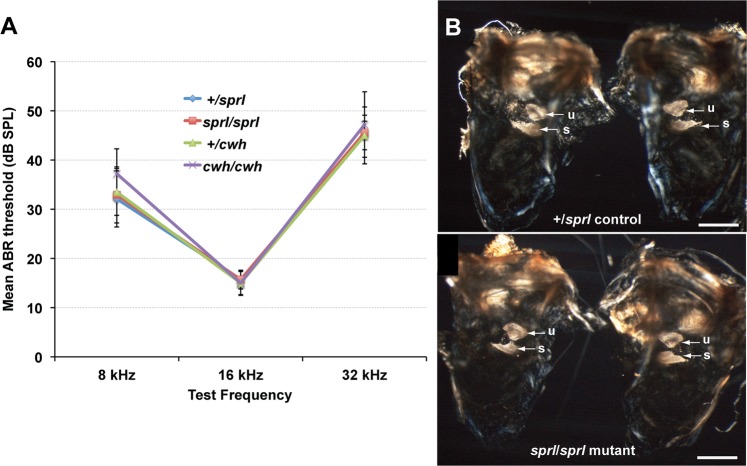
Inner ear-related phenotypes of *sprl*/*sprl* and *cwh*/*cwh* mutant mice. (**A**) Mutant mice have normal hearing thresholds. Average ABR thresholds for 8, 16, and 32 kHz stimuli and associated standard error bars are shown for +/*sprl* (5f, 2 m), *sprl*/*sprl* (8f), +/*cwh* (6f, 3 m), and *cwh*/*cwh* (4f, 4 m) mice at 4–8 weeks of age. The thresholds were not statistically significantly different from one another, and all were in the normal range for young, good-hearing mice. (**B**) Mutant mice have intact otoconial membranes. Left and right cleared inner ears from *Zpld1*^+/*sprl*^ controls (top) and *Zpld1*^*sprl*/*sprl*^ mutant (bottom) adult mice were exposed to polarized light to accentuate the calcium carbonate crystals (indicated by arrows) embedded in the otoconial membranes of the utricle (u) and saccule (s). No gross structural abnormalities were seen in the inner ear of the *sprl*/*sprl* mice compared with the +/*sprl* control mouse. Scale bars, 0.75 mm.

Because vestibular dysfunction is often accompanied by cochlear dysfunction, hearing acuity in the mutant mice was examined in more detail by auditory brainstem response (ABR) analysis. Mice with the *cwh* mutation were tested at four weeks (4 +/*cwh* and 3 *cwh*/*cwh*), eight weeks (5 +/*cwh* and 5 *cwh*/*cwh*), and thirty weeks of age (1 +/*cwh* and 1 cwh/*cwh*). Mice with the *sprl* mutation were tested at five and seven weeks of age (7 +/*sprl* and 7 *sprl*/*sprl*). The ABR thresholds of *cwh*/*cwh* and *sprl*/*sprl* mutant mice did not vary at the different test ages and did not differ from those of heterozygous controls at 4–8 weeks of ag (Fig. 1A). Thresholds of the one +/*cwh* and one *cwh*/*cwh* mice tested at 30 weeks of age were not significantly different from those shown in Fig. 1A. Thresholds of all mice were in the normal range for age-matched +/+ mice of the parental C57BL/6J (B6) strain^20^ (Fig. S1).

Because many mouse mutations affecting otoconia formation cause vestibular dysfunction with behavioral consequences^12^, otolith organs were carefully examined in both *cwh*/*cwh* and *sprl*/*sprl* mutant mice. Microscopic examination with polarized light revealed normal appearing otoconia in both the saccule and the utricle of *sprl*/*sprl* (Fig. 1B) and *cwh*/*cwh* (Fig. S2) mutant mice. In swim tests, 1.5-month-old +/*cwh* mice (n = 6) manifested normal swimming behaviors, whereas all age-matched *cwh*/*cwh* mutant mice (n = 4) swam in circles at the water’s surface (*sprl*/*sprl* mutant mice were not tested). The ability to remain at the surface indicates the presence of some degree of gravity receptor function, consistent with the observation of intact otoconial membranes in mutant mice; however, swimming in circles at the surface suggests semicircular canal dysfunction. Histological examinations of inner ear cross-sections from mutant and control mice revealed no gross morphological abnormalities in the cochlea or vestibular organs. Remnants of the cupula could be seen in the crista ampullaris of both mutant and control mice, but the delicate nature of this extracellular membrane precluded the ability to make comparative assessments of its morphology. The gelatinous substance that forms the cupula frequently shrinks during fixation and dehydration processes.

### Genetic mapping and identification of *Zpld1* as the mutated gene

Linkage associations of mouse circling behavior (indicating homozygosity for the recessive *cwh* and *sprl* mutant alleles) with genotypes of polymorphic chromosomal markers distributed throughout the genome were used to genetically map the mutations. A linkage backcross of (B6(129P2)-*cwh* homozygote × CAST/EiJ) F1 hybrids (*cwh*/+) to B6(129P2)-*cwh* homozygotes produced 38 N2 mice with clearly identifiable circling behaviors (inferred *cwh*/*cwh* genotype), which were used to map *cwh* to Chr 16 between microsatellite markers *D16Mit39* (43.5 Mb position on Chr 16, GRCm38) and *D16Mit64* (57.5 Mb). A linkage intercross of (B6(129S4)-*sprl* homozygote x CAST/EiJ) F1 hybrids (*sprl*/+) produced 51 F2 progeny with clearly identifiable circling behaviors (inferred *sprl*/*sprl* genotype), which were used to map *sprl* to Chr 16 between *D16Mit14* (51.4 Mb) and *D16Mit64* (57.5 Mb) and non-recombinant with *D16Mit30* (54.0 Mb*)*. The similar phenotypes and overlapping chromosomal map positions strongly suggested that *cwh* and *sprl* are mutations of the same gene.

**Figure 2:**
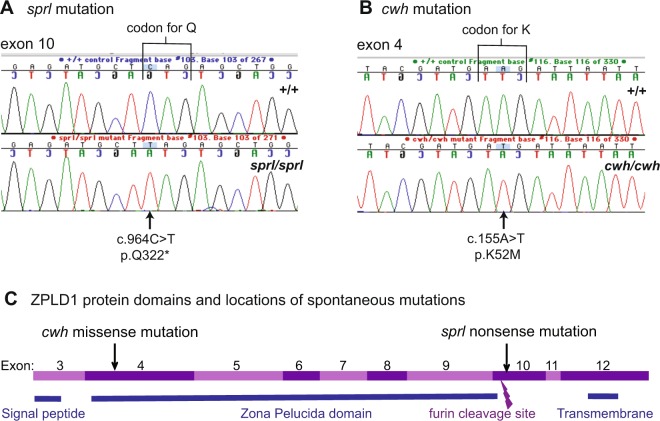
The *sprl* and *cwh* mutations occur in protein coding regions of the *Zpld1* gene. (**A**) *sprl* is a nonsense mutation in exon 10. Sequence chromatograms of *Zpld1* exon 10 in +/+ and *sprl*/*sprl* mice show that the *sprl* mutation is a single base pair change (c.964C > T) that alters the CAG codon for glutamine (Q) to a TAG stop codon at residue 322 of the protein (p.Q322*). (**B**) *cwh* is a missense mutation in exon 4. Sequence chromatograms of *Zpld1* exon 4 in +/+ and *cwh*/*cwh* mice show that the *cwh* mutation is a single base pair change (c.155A > T) that alters the AAG codon for lysine (K) to the ATG codon for methionine (M) at residue 52 of the protein (p.K52M). (**C**) Structural diagram of the ZPLD1 protein derived from the Ensembl genome browser. Protein-coding regions of the *Zpld1* gene encoded by exons 3–12 are shown as alternating pink and purple bands, with corresponding protein domains shown below by blue bars. Arrows indicate the sites of the *cwh* and *sprl* mutations. A lightning bolt symbol indicates the furin cleavage site.

The 6.1 Mb candidate region for the *sprl* mutation (Chr 16: 51.4–57.5 Mb) contains 25 protein coding genes. To identify which of these genes is altered by the mutation, we undertook whole-exome sequencing analysis, comparing exon and adjacent splice site sequences from genomic DNA of B6(129S4)-*sprl*/*sprl* mice with those of the B6 reference DNA sequence, paying particular attention to sequence differences in the genetically determined candidate region. Within this region, we identified seven high quality sequence differences between B6-+/+ DNA and B6(129S4)-*sprl*/*sprl* DNA that occur within exons or flanking splice sites. Six of the DNA sequence differences were previously known inbred strain polymorphisms. The remaining difference was unique and predicted to be highly detrimental. A single base pair change in exon 10 of the zona pellucida like domain containing 1 gene (*Zpld1*) was consistently detected in all sequencing reads from the *sprl*/*sprl* mutant compared with the B6-+/+ control, and this difference was predicted to introduce a premature stop codon and thus have a high impact on protein function. The single nucleotide change (c.964 C > T) identified in exon 10 of *Zpld1* changes the CAG codon for glutamine to a TAG stop codon at residue 322 (p.Q322*) of the protein (Fig. 2A). To confirm causality of this mutation, we sequenced PCR-amplified DNA products from additional mutants (*sprl*/*sprl*) and B6 controls (+/+) and obtained consistent correspondence of *Zpld1* genotypes with vestibular phenotypes.

Because the genetic map positions and associated phenotypes of *cwh* and *sprl* suggest that they may be alleles of the same gene, we screened DNA from *cwh*/*cwh* mice for possible *Zpld1* mutations. We sequenced all 12 exons and flanking regions of the *Zpld1* gene and discovered a single base pair change in exon 4 compared with the B6 reference sequence. To confirm the causative nature of this mutation, we sequenced PCR-amplified DNA products from additional mutants (*cwh*/*cwh*), heterozygotes (+/*cwh*) and B6 controls (+/+), and the inferred *cwh* genotypes of all mice agreed with their *Zpld1* genotypes. The single nucleotide change in *Zpld1* cDNA of the *cwh* mutation is predicted to have a negative impact on protein function. The mutation (c.155 A > T) changes the AAG codon for lysine to the AUG codon of methionine at residue 52 (p.K52M) of the protein (Fig. 2B). This missense mutation of a highly conserved lysine residue is predicted to be damaging by the PolyPhen-2 software tool with a score of 0.998.

The positions of the *cwh* and *sprl* mutations in ZPLD1 relative to its protein domains and furin cleavage site are shown in Fig. 2C. The *cwh* missense mutation occurs in exon 4, which encodes part of the zona pelucida domain of the protein. The *sprl* nonsense mutation in exon 10 is predicted to stop translation immediately past the furin cleavage site, eliminating the C-terminal region of the protein including the transmembrane domain.

### Localization of *Zpld1* mRNA expression in the inner ear

**Figure 3:**
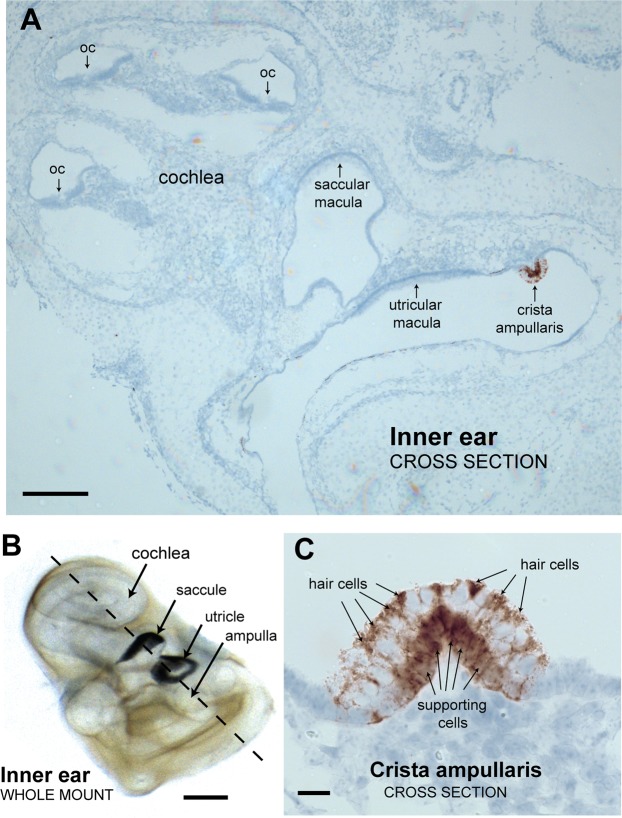
*Zpld1* mRNA expression in the inner ear is primarily restricted to cells of the crista ampullaris. (**A**) *In situ* hybridization results showing a cross section of an inner ear from a newborn B6 mouse with areas of positive *Zpld1* expression stained brown with a blue counter stain (Hematoxylin). Sensory epithelia with overlying acellular membranes are indicated by arrows, and include the organ of Corti (oc) in the cochlea, the saccular and utricular maculae, and the crista ampullaris. *Zpld1* expression was detected only cells of the crista ampullaris. Scale bar, 200 μm. (**B**) A cleared, whole mount preparation of an inner ear from a newborn mouse positioned to match the orientation of the cross section shown in panel A. Otoconial crystals block microscope illumination from below, giving the saccular and utricular maculae a dark appearance. The dashed line indicates the corresponding plane of dissection. Scale bar, 500 μm. (**C**) Higher magnification of a crista ampullaris shows that *Zpld1* appears to be expressed in both hair cells and supporting cells. Positive expressing cells at the crista surface were presumed to be hair cells, and positive expressing cells near the base were presumed to be supporting cells. Scale bar, 20 μm.

We examined four inner ears from newborn B6 mice for *Zpld1* expression. Our mRNA *in situ* hybridization assays of inner ear cross sections detected *Zpld1* expression only in cells of the crista ampullaris, as illustrated in the inner ear cross-section shown in Fig. 3A. For orientation, the position of this section in a whole mount of the inner ear is shown in Fig. 3B. Cristae in all three canals showed the same *Zpld1* expression pattern. For example, the crista cross-section shown in Fig. 3A and the crista cross-section shown at higher magnification in Fig. 3C are from different semicircular canal ampullae of the same inner ear. That *Zpld1* is highly expressed in cells that synthesize molecular components of the cupula, but not in cells that produce components of the tectorial or otoconial membranes, explains why semicircular canal function is impaired in mutant mice but cochlear and otolithic organ functions are not. Because *Zpld1* expression appears primarily restricted to cells of the crista ampullaris, it may provide a useful marker for these cells.

Our attempts to localize the ZPLD1 protein in histological sections of mouse inner ears using multiple commercially available antibodies failed because of problems with non-specific reactivity and the degenerated condition of the gelatinous cupula (the predicted site of ZPLD1 expression) resulting from the tissue preparation process.

### Refined tests of vestibular function – VsEP and VOR measurements

The circling behavior of mutant mice suggests vestibular dysfunction; however, no histological abnormalities were detected in the vestibular organs (although structural abnormalities of the fragile ampullary cupula could not be reliably evaluated). Electrophysiological methods, therefore, were used to directly test vestibular function in the mutant mice. Vestibular sensory evoked potential (VsEP) measurements were used to evaluate gravity receptor (otolith) function, and vestibulo-ocular reflex (VOR) and optokinetic reflex (OKR) tests were used to evaluate semicircular canal function. Mice were sent from the Jackson Laboratory to the University of Nebraska, Lincoln (UNL), for the VsEP and VOR tests and were also examined for ABR. VsEP and ABR tests were performed on mice at 1.5 to 8 months of age and VOR/OKR testes were done on mice at 11–13 months of age.

**Figure 4:**
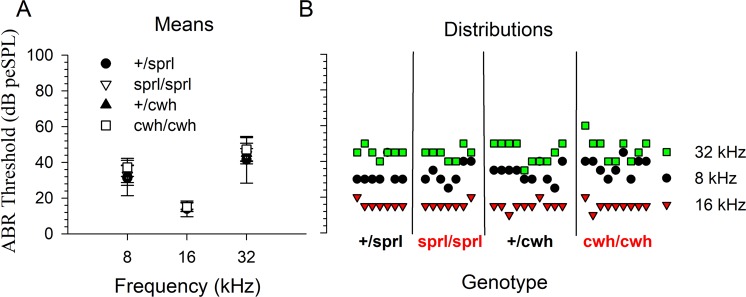
Normal ABR threshold means and individual distributions were observed in mice tested at UNL. (**A**) Mean ABR thresholds at 8, 16 and 32 kHz (error bars = standard deviation). (**B**) Distribution of individual ABR thresholds for each genotype at each frequency tested.

The hearing thresholds for mice of all four genotypes examined (+/*sprl*, *sprl*/*sprl*, +/*cwh*, and *cwh*/*cwh*) did not differ from each other and were in the normal range for good hearing mice (Fig. 4A), confirming earlier results obtained at The Jackson Laboratory (Fig. 1A) and demonstrating that there was no major deterioration of auditory function with transport of animals to UNL. The distributions of individual ABR thresholds across genotypes were comparable (Fig. 4B).

#### VsEP findings

**Figure 5:**
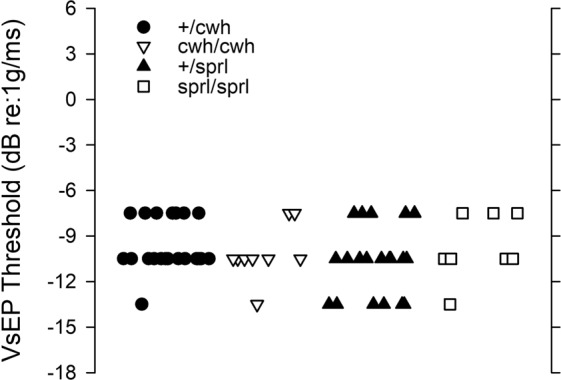
Vestibular sensory thresholds were normal in *cwh*/*cwh* and *sprl*/*sprl* mutants. VsEP thresholds for individual animals of the four genotypes studied are shown. There were no differences in mean threshold across genotypes (ANOVA) and thresholds were within normal range for all genetic variants (all less than −4.5 dB re:1 g/ms).

**Figure 6:**
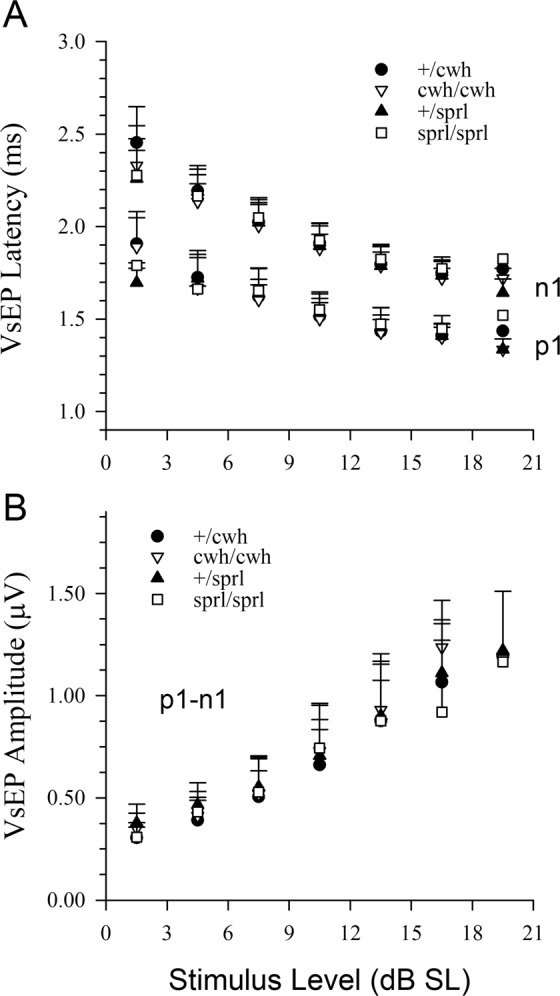
VsEP amplitudes and response activation timing were normal in *cwh*/*cwh* and *sprl*/*sprl* mutants. Mean ± standard deviation are shown. (**A**) Latencies of p1 and n1 are shown as a function of stimulus level above threshold (sensation level) in dB SL. There was no evidence of differences in the input-output (IO) relationships for VsEP latencies. Timing of response activation was indistinguishable between genotypes. (**B**) Amplitudes as a function of stimulus level. Amplitudes of p1-n1 are shown as a function of stimulus level above threshold (dB SL) for each genotype. There were no significant differences between genotypes (rmMANOVA p1-n1, p2-n1). Stimulus levels of 7.5. 10.5 and 13.5 were evaluated statistically since these gave the maximum sample sizes for groups.

There were no detectable differences in VsEP thresholds between homozygotes and heterozygotes for mice with the *cwh* or *sprl* mutations (Fig. 5). There were also no detectable differences in VsEP response activation latencies (p1, n1 & p2) or amplitudes (p1-n1, p2-n1) at the highest stimulus level (+6 dB re:1 g/ms) across genotypes (data not shown). Latencies decreased (Fig. 6A) whereas amplitudes increased (Fig. 6B) systematically with increasing stimulus level in all animals and these input-output-functions were indistinguishable across mutant genotypes and corresponding heterozygous animals.

#### VOR findings

**Figure 7:**
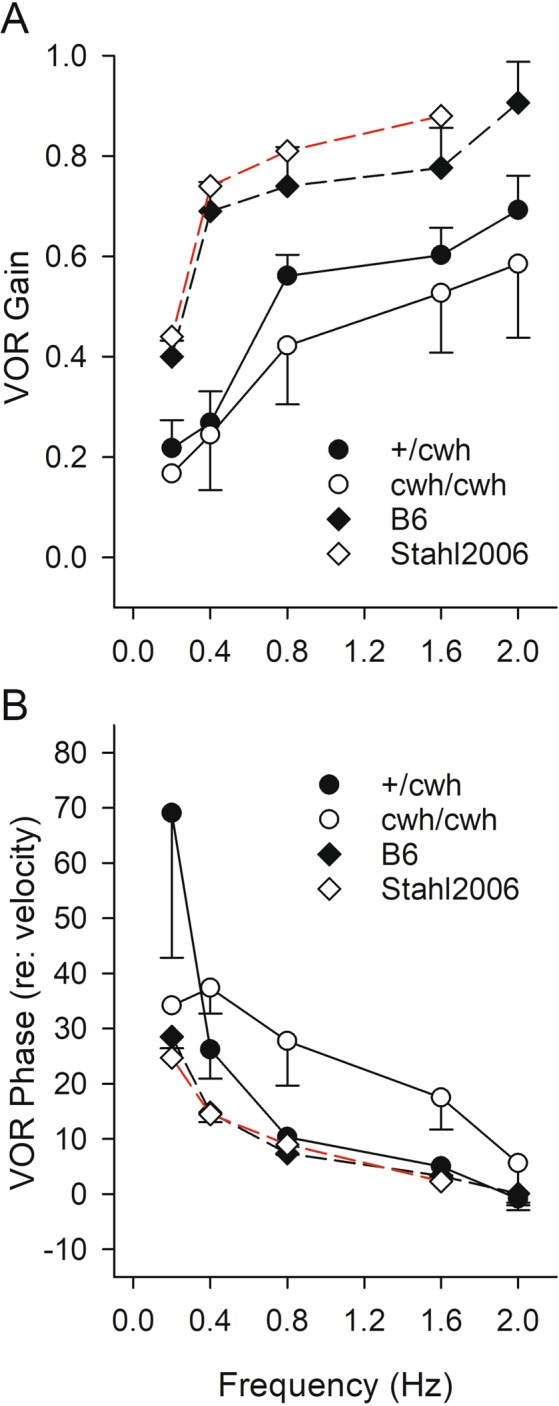
VOR gain and phase in *cwh* genotypes. Means ± standard errors are shown. (**A**) VOR gain increased as a function of rotational frequency for both heterozygotes (+/*cwh*) and homozygotes (*cwh*/*cwh*) and there was no significant difference between the two gene variants. At 0.2 Hz, results were obtained from only one *cwh*/*cwh* animal. Mean values for both genotypes were also somewhat lower than values reported for internal +/+ control B6 mice (n = 3, 3–4 months of age) and comparative values previously reported for 8–14 month-old normal B6 mice (Stahl *et al*., 2006). (**B**) VOR phase decreased systematically with increasing frequency for both genotypes. Mean values were comparable for both heterozygous and homozygous *cwh* genotypes and for +/+ controls (both internal and previously published B6 mice).

**Figure 8:**
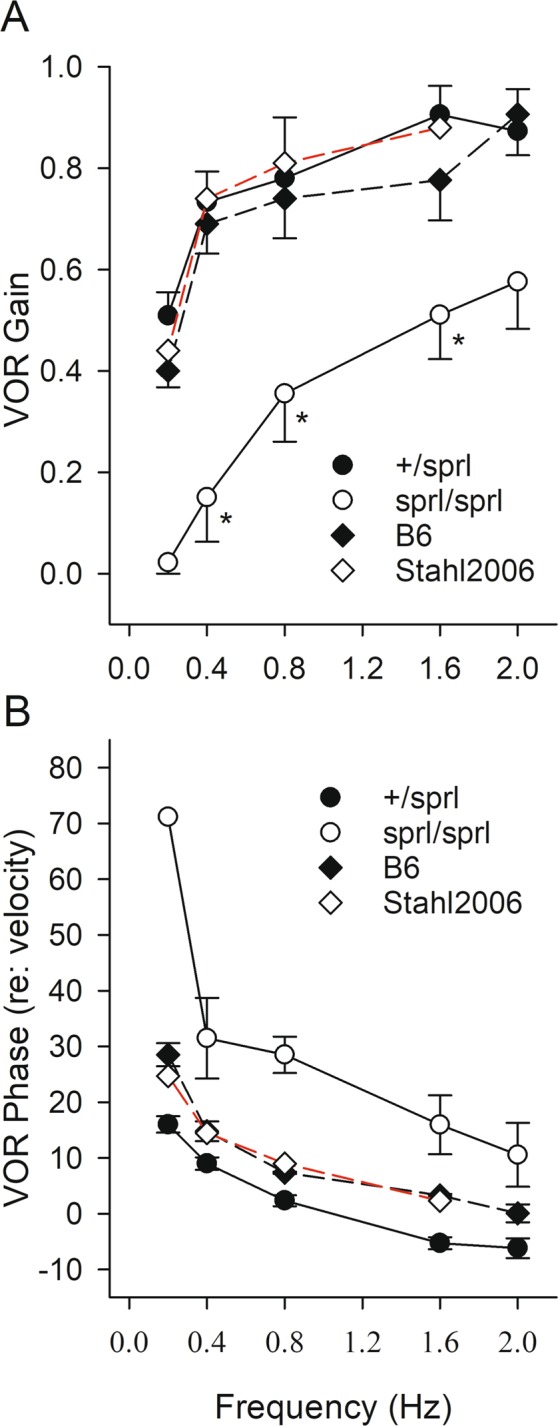
VOR gain and phase in *sprl* genotypes. Means ± standard errors are shown. (**A**) VOR gain for *sprl* homozygotes (*sprl*/*sprl*) and heterozygotes (+/*sprl*) are plotted as a function of rotational frequency. Gain increased with increasing frequency for both genotypes. However, mean VOR gain for *sprl*/*sprl* animals was significantly reduced compared to +/*sprl* animals (*, post hoc p < 0.031). At 2 Hz, the difference in means did not reach significance (p = 0.052). Also plotted are mean VOR gains from internal +/+ control B6 mice (n = 3, 3–4 months of age) and those previously reported by Stahl and colleagues for normal B6 mice (Stahl *et al*., 2006). Mean gains for +/*sprl* animals were comparable to the normal B6 mice, whereas gains for *sprl*/*sprl* mice were much lower. (**B**) VOR phase decreased systematically with increasing rotational frequency. Mean values for heterozygotes were similar to those of +/+ control B6 mice. Mean phases for *sprl*/*sprl* mice were significantly larger indicating relatively sluggish compensation in *sprl*/*sprl* mutants. At 0.2 Hz, gains for *sprl*/*sprl* animals were near zero and as a result, phase estimates were unreliable and thus not included.

For all genotypes, mean values for VOR gain increased with increasing angular frequency thus following a generally normal mammalian pattern (Figs 7 and 8, repeated measures MANOVA p = 3.0 × 10^−6^). On average across frequencies, gain was approximately 16% lower (although not significantly) in homozygous *cwh*/*cwh* animals compared to heterozygous +/*cwh* mice (Fig. 7A), whereas gain was substantially and significantly reduced by about 59% on average in homozygous *sprl*/*sprl* mutants compared to heterozygous +/*sprl* controls (Fig. 8A, repeated-measures MANOVA p = 0.006, Bonferroni post hoc p = 0.007). Although not significantly different than the +/*cwh* heterozygotes, VOR gains for *cwh*/*cwh* homozygotes were nonetheless abnormally low compared with +/+ B6 mice and significantly lower than heterozygous +/*sprl* controls (Bonferroni post hoc p = 0.029). There was no significant difference in gains between *cwh*/*cwh* and *sprl*/*sprl* mutants. These findings indicate a substantial VOR weakness in homozygotes of both *cwh* and *sprl* gene variants. Notably, gains for *cwh* heterozygotes were also lower than those typically reported for normal B6 mice (e.g., see^21,22^), thus explaining the finding of no difference between *cwh* heterozygotes and homozygotes. In contrast, gains for +/*sprl* heterozygotes were comparable to normal B6 mice, and VOR gains for *sprl*/*sprl* homozygotes were significantly reduced compared to heterozygous +/*sprl* animals (p = 0.007). VOR phase systematically decreased with increasing frequency in all genotypes (Figs 7B and 8B) and there were no significant differences in the profiles. On the whole, these findings indicate that mutant forms of the *Zpld1* gene encoding the ZPLD1 protein known as cupulin^17,19^ lead to significant vestibular semicircular canal dysfunction and that cupulin therefore plays a significant role in normal ampullary sensory function.

#### Optokinetic response (OKR) findings

Robust OKRs were seen in all *cwh* and *sprl* genotypes in response to stripe rotation stimuli. There was no significant effect of genotype on OKR gain. The mean values for OKR gain were somewhat lower for all treatment groups (clockwise −0.67 ± 0.18 and counter clockwise 0.66 ± 0.25) compared to gains reported elsewhere during head rotation in the light^21,22^. The values are reasonable however for 10 degrees/s velocity due to the absence of a vestibular contribution to the ocular response since animals were stationary during the OKR testing. Based on the work of Stahl and colleagues^21,22^, OKR gain values on the order of 0.5 to 0.6 would be anticipated for drum velocities of 10 degrees/s. OKR results obtained in the *cwh* and *sprl* mutant strains here are thus consistent with normal OKR function as measured in B6 mice. These findings rule against a significant role for central oculomotor circuits in the functional loss indicated by VOR findings.

### Phenotype of a genetically engineered *Zpld1* knockout mutation

A *Zpld1* knockout mutation (*Zpld1*^*em1(IMPC)J*^), generated by the Knockout Mouse Phenotyping Program (KOMP^2^) at The Jackson Laboratory, was recently made publicly available. We obtained some of these mice and found that the homozygous mutant mice (here designated *Zpld1*^*−*/*−*^), like *Zpld1*^*cwh*/*cwh*^ and *Zpld1*^*sprl*/*sprl*^ mice, had normal hearing. Surprisingly, however, none of the 26 *Zpld1*^*−*/*−*^ mice examined displayed circling behavior, whereas 100% of *Zpld1*^*cwh*/*cwh*^ mice and 48% of *Zpld1*^*sprl*/*sprl*^ mice did (Table 1). We reasoned that the difference in behavioral phenotypes between the knockout mutation and the spontaneous *cwh* and *sprl* mutations must be due to differences either in their strain backgrounds or in the nature of their mutations.

**Table 1:** Prevalence of circling behavior in mice with different *Zpld1* genotypes.

	*Zpld1* genotypes					
	+/*cwh*	*cwh*/*cwh*	+/*sprl*	*sprl*/*sprl*	−/−	*−*/*sprl*
Total number of mice	37	25	12	75	26	63
Number that circle	1	25	0	36	0	17
Penetrance of circling	3%	100%	0%	48%	0%	27%

All mice examined were on a primarily C57BL/6 strain background. *Zpld1* genotypes of all mice were determined by DNA analysis.

The knockout mutation was generated on the C57BL/6NJ strain, whereas the *sprl* mutation originated on the B6.129S4-*Ccl2*^*tm1Rol*^/J congenic strain, and the *cwh* mutation originated on the B6.129P2-*Nos2*^*tm1Lau*^/J congenic strain. These two congenic strains are genetically very similar to the B6 strain except for distal regions of Chr 11 surrounding the introgressed *Ccl2* and *Nos2* genes, which came from the 129S4/SvJae-derived and 129P2/OlaHsd-derived ES cells in which the *Ccl2*^*tm1Rol*^ and *Nos2*^*tm1Lau*^ alleles were generated, respectively. It was therefore possible that genetic factors in the 129 strain-derived distal Chr 11 regions, if retained, might modify the *sprl* and *cwh* mutant phenotypes. To test for this possibility, we genotyped 44 SNPs spanning Chr 11 that differed between B6 and 129 strains and found only B6 alleles at all SNP loci in both the *cwh* and *sprl* strains, indicating that the 129-derived congenic regions of the parental strains were eliminated during the derivations of these strains. Thus, all three *Zpld1* mutations are on essentially identical C57BL/6 strain backgrounds, making it highly unlikely that strain background differences account for the phenotypic differences.

Unfortunately, we were unable to perform VsEP, VOR, and OKR tests on the recently available *Zpld1* knockout mice because the laboratory of Dr. Sherri Jones (University of Nebraska, Lincoln), in which these tests had been performed on *Zpld1*^*sprl*^ and *Zpld1*^*cwh*^ mutant mice, had since been closed and the person performing the tests (Sarath Vijayakumar) had subsequently moved to Creighton University (Omaha, NE), where testing facilities would have to be set up anew. We therefore decided to indirectly test whether the *Zpld1* knockout mutation has an effect on vestibular function by combining it with the *sprl* mutation to produce *Zpld1*^*−*/*sprl*^ compound heterozygotes, which then would be examined for vestibular-related motor behavior. An obvious circling behavior was observed in 27% of the *Zpld1*^*−*/*sprl*^ mice (Table 1, Supplementary Video 3), indicating that the *Zpld1*^−^ knockout allele does indeed contribute to balance dysfunction in these mice. That *Zpld1*^*−*/*sprl*^ trans heterozygous mice exhibit circling behavior also demonstrates non-complementation of the *sprl* and *Zpld1*^−^ alleles, providing further evidence that *Zpld1* is the causative gene underlying the phenotype of *sprl* mutant mice.

## Discussion

The ZPLD1 protein (also known as cupulin) was recently found to be a component of the cupula in the inner ear of salmon^19^. Here, we provide gene expression evidence indicating that ZPLD1 is also a component of the mouse cupula and show that mutations of *Zpld1* cause semicircular canal dysfunction, which often results in hyperactive circling behavior of mutant mice. *Zpld1*^*sprl*/*sprl*^ and *Zpld1*^*cwh*/*cwh*^ mutant mice exhibit normal hearing (as shown by ABR thresholds, Figs 1A, 4) and gravity receptor function (as shown by VsEP responses, Figs 5 and 6), but have pronounced semicircular canal dysfunction (as shown by VOR responses, Figs 7 and 8). In wildtype B6 mice, we detected *Zpld1* mRNA expression specifically in the sensory hair cells and supporting cells of the crista ampullaris (Fig. 3), which synthesize components of the cupula. *Zpld1* expression was not detected in the sensory epithelial cells of the utricle, saccule, and cochlea; therefore, the otoconial and tectorial membranes that overlie these cells must lack ZPLD1. These membranes, however, contain other ZP domain-containing proteins, the alpha and beta tectorins^17^, which may provide a similar function.

The finding of reduced VOR gain, which is a measure of semicircular canal function, in mutant mice of the present study is consistent with *Zpld1* mRNA expression being detected only in cells of the crista ampullaris. The findings demonstrate that *Zpld1* plays an important role in cupular and in turn epithelial receptor function in the crista. Gain was reduced generally across frequency; a finding that could indicate that the defect may reduce transfer of stimulus from the cupula to the epithelium. This could result for example from changes (e.g., increase) in the mechanical impedance of the cupular apparatus including altered cupular stiffness, altered adhesion to the ampular wall or reduced coupling to the epithelial receptors.

The reduced VOR gain in the +/*cwh* heterozygous animals is interesting in that it may point to an unusual phenotype that persists and is not completely rescued by the wildtype allele in heterozygotes. We hypothesize that the missense nature of the *cwh* mutation causes a toxic gain-of-function malformation of the ZPLD1 protein that is disruptive to the matrix structure and function of the cupula, even in the presence of the wildtype protein in heterozygotes. Although all +/*cwh* heterozygotes exhibited a reduced VOR gain (Fig. 7A), only one out of 37 (3%) showed circling behavior (Table 1). Behavioral indicators such as circling may not be sufficiently sensitive to resolve the residual vestibular deficit in *cwh* heterozygotes.

All *cwh*/*cwh* mice exhibit circling behavior and all +/*cwh* mice exhibit reduced VOR gain. In contrast, only about half of *sprl*/*sprl* mice circle and no +/*sprl* mice show reduced VOR gain. The different effects of the *cwh* and *sprl* variants of *Zpld1* are interesting in that they provide an additional example of how distinct genetic variants of the same gene can lead to functional losses of different magnitudes. Well-known, perhaps extreme examples of this for the inner ear include the various mutations of the cadherin 23 gene, where the magnitude and time course of functional loss ranges from profound loss of both vestibular and auditory function at birth^23–27^ to age related hearing loss with no accompanying vestibular deficit^28^.

The *sprl* mutation is predicted to eliminate the transmembrane domain (TMD) and the external hydrophobic patch (EHP) of the ZPLD1 protein, which lies between the furin cleavage site and the TMD. The TMD domain is necessary for the initial anchoring of ZP protein precursors in the plasma membrane for proteolytic processing. The EHP and the internal hydrophobic patch (IHP), which is located within the ZP domain, are duplicate short hydrophobic motifs conserved in ZP domain proteins^15^. According to a proposed mechanism, interaction between the EHP and the IHP blocks the assembly property of the ZP domain, thereby preventing premature polymerization until proteolytic processing removes the EHP and activates the mature protein^29^. The loss or absence of the EHP before assembly and processing at the plasma membrane could cause premature intracellular polymerization of the nascent ZP protein, which then would disrupt intracellular vesicular transport and lead to its accumulation in the ER. In support of this possibility, a cell culture study of a recombinant zona pellucida glycoprotein 3 (ZP3) construct that was truncated immediately after the consensus furin cleavage site (analogous to the predicted *sprl* truncation of ZPLD1) failed to secrete ZP3 into the culture medium, and un-cleaved nascent ZP3 accumulated in the ER^30^. The *sprl* mutation likewise may cause a deleterious build up of prematurely polymerized protein that accumulates in the ER of cells in the crista. In homozygous mutant mice this inability to properly process ZPLD1 could lead to cellular stress and diminished function of the ZPLD1-producing sensory cells in the crista.

As mentioned in the Results, we were unable to test *Zpld1*^*−*/*−*^ knockout mice for VsEP, VOR, and OKR and so could not definitively assess their vestibular function. Surprisingly, in contrast to *Zpld1*^*cwh*/*cwh*^ and *Zpld1*^*sprl*/*sprl*^ mutant mice, the *Zpld1*^*−*/*−*^ knockout mice did not exhibit circling behavior, suggesting that the spontaneous *cwh* and *sprl* mutations have more severe effects on balance function than does ZPLD1 deficiency alone. To explain the differences in mutation effects, we hypothesize that the the *Zpld1*^*cwh*^ missense mutation produces a toxic, gain-of-function protein that interferes with other components of the cupula and disrupts the overall matrix structure, which could have a more negative effect on function than the loss of ZPLD1 caused by the knockout mutation. We hypothesize that the *Zpld1*^*sprl*^ mutation encodes a truncated protein whose defective cellular processing may impair hair cell as well as cupula function, which also could result in a more negative effect on vestibular function than the *Zpld1* knockout mutation. None of the mice homozygous for the *Zpld1*^*−*^ knockout allele, which completely lack the ZPLD1 protein, exhibited circling behavior; however, when the *Zpld1*^−^ allele was combined with the *sprl* allele, 27% of the compound heterozygotes (*Zpld*^*−*/*sprl*^) did show circling behavior (Table 1). These results provide indirect evidence that presence of the wildtype ZPLD1 protein is necessary for normal vestibular function. *Zpld1*^*−*/*−*^ mice likely exhibit semicircular canal dysfunction that, although not severe enough to elicit an easily observable circling behavior, may be detectable by more sensitive means, analogous to our findings for *Zpld1*^+/*cwh*^ heterozygotes, which consistently show abnormal VORs but only rarely exhibit circling behavior.

In summary, we provide evidence that ZPLD1 is an extracellular protein component of the cupula in mice, that *Zpld1* mRNA expression in the inner ear is primarily restricted to cells of the crista ampullaris, and that *Zpld1* mutations lead to vestibular but not auditory dysfunction. *Zpld1* mutant mice may provide models for some types of human peripheral vestibular disorders, most of which have unknown etiologies. They also may be useful for studying the physiological and behavioral consequences of semicircular canal dysfunction without the confounding influence of otolith organ defects.

## Methods

### Mice

All mice used in this study originated from and are maintained in the Research Animal Facility of The Jackson Laboratory (Bar Harbor, ME). Some post-weaning mice were shipped to the University of Nebraska, Lincoln, Nebraska for additional experimental analyses. All procedures involving the use of experimental mice were approved by the Institutional Animal Care and Use Committees at The Jackson Laboratory and the University of Nebraska. All methods used in the study were performed in accordance with the guidelines and regulations of the U.S. National Institutes of Health (NIH) Office of Laboratory Animal Welfare (OLAW) and the Public Health Service (PHS) Policy on the Humane Care and Use of Laboratory Animals.

The spontaneous spiral (*sprl*) mutation arose in a colony of B6.129S4-*Ccl2*^*tm1Rol*^/J mice at The Jackson Laboratory (JAX). The *Ccl2* knockout allele of this congenic strain was created in 129S4 ES cells and then backcrossed onto the C57BL/6J (B6) strain. Accordingly, the derivative strain carrying the *sprl* mutation is officially designated B6(129S4)-*Zpld1*^*sprl*^/Kjn (JAX Stock No. 28920). The *Ccl2* knockout allele was removed from this strain during its derivation. The spontaneous circler with hearing (*cwh*) mutation arose in a colony of B6.129-N*os2*^*tm1Lau*^/J strain mice at JAX. The *Nos2* gene knockout allele of this congenic strain was created in 12P2/OlaHsd ES cells and then backcrossed onto the C57BL/6J strain. Accordingly, the derivative strain carrying the *cwh* mutation is officially designated B6(129P2)-*Zpld1*^*cwh*^/Kjn (JAX Stock No. 6123). The *Nos2* knockout allele was removed from this strain during repeated backcross-intercross breeding to C57BL/6J, which was done until generation NE5 prior to maintaining by sibling intercross. Mutant *sprl*/*sprl* and *cwh*/*cwh* mice are fertile and able to breed.

We obtained mice with a *Zpld1* knockout allele from the Knockout Mouse Phenotyping Program (KOMP^2^) at JAX. The knockout mutation was generated using CRISPR technology, and the strain carrying the mutation is designated C57BL/6NJ-*Zpld1*^*em1(IMPC)J*^/Mmjax (MMRRC Stock No. 43719-JAX). The targeted alteration resulted in the deletion of 477 bp, which should result in the deletion of exon 4 and 256 bp of flanking intronic sequence, including the splice acceptor and donor. The mutation is predicted to cause a change of amino acid sequence after residue 35 and early truncation 25 amino acids later, thus resulting in a complete loss of function (https://www.jax.org/strain/031591).

### Genetic mapping and whole exome sequencing

To genetically map the *sprl* and *cwh* mutations, individual DNA samples from linkage cross mice were typed for multiple MIT microsatellite markers located throughout the mouse genome. Previously described PCR methods^31^ were used to genotype the chromosomal markers, which were then analyzed for co-segregation with the mutant phenotype (circling behavior). PCR primer pairs designed to amplify specific markers were purchased from Integrated DNA Technologies (Coralville, IA, USA).

Whole-exome sequencing was used to identify the *sprl* mutation in the *Zpld1* gene. Purified genomic DNA from *sprl*/*sprl* mice was used to create a library for whole-exome sequence capture. Exon sequences from this library were compared with those of the C57BL/6J reference sequence. DNA purification, library construction, deep sequencing, and data quality control were performed by the Jackson Laboratory’s Next Generation Sequencing service, and data analysis and annotation were performed by the Computational Sciences-Biostatistics service.

### **DNA analysis of the*sprl*and*cwh*mutations**

PCR primers used to amplify the exon 10 region of the *Zpld1* gene containing the *sprl* mutation were 5′-CTTTCCAAACGGCCATAAAG-3′ (forward) and 5′-CAGGGTAAATGGGAAACTGC-3′ (reverse) and expected to produce a product size of 330 bp. PCR primers used to amplify the exon 4 region of the *Zpld1* gene containing the *cwh* mutation were 5′-TCGTTCACCTGCCCTCTACT-3′ (forward) and 5′-TTTCGTGAAACTGGAGCTTG-3′ (reverse) and expected to produce a product size of 354 bp. The expected PCR product sizes were confirmed on a 3.5% agarose gel, and PCR products were purified with the QIAquick PCR Purification Kit (Qiagen Inc., Valencia, CA). The same primers used for PCR amplification were used to sequence the purified PCR products on an Applied Biosystems 3700 DNA Sequencer with an optimized Big Dye Terminator Cycle Sequencing method. Typical DNA sequencing results to identify *sprl* and *cwh* mutant alleles for genotyping are shown in Fig. 2.

### Swim test to assess vestibular function

A 2L wide-top beaker is filled with sterile water to a depth of at least 15 cm to allow free movement and swimming of the mouse. The water is warmed to room temperature before testing, and an empty mouse box (with paper towels on the bottom) is pre-warmed on a slide warmer so that the bottom of the box is 30 °C. The mouse to be tested is removed from its home box and lowered carefully into the room-temperature water and then observed for a maximum of 1 minute to assess its swimming ability. If the mouse displays continuous underwater tumbling or any other distressing behavior, it is rescued immediately. After removal from the water tank, the mouse is gently patted dry with paper towels and placed on dry paper towels in the pre-warmed mouse box. Before being returned to the mouse room, mice are kept under observation in the heated box until they are completely dry and exhibit normal behavior.

### ZPLD1 immunohistochemistry

Adult mice were transfused transcardially with 4% paraformaldehyde in 0.1M phosphate buffer. Inner ears were removed and flushed with the same fixative, and post-fixed overnight at 4 °C. After fixation, inner ears were placed in decalcification solution (0.35M EDTA, 0.1M sodium phosphate, pH 7.4) for 1 week at 4 °C, rinsed in phosphate buffer, cryoprotected with buffered sucrose, and frozen in OCT compound. Cryostat sections (10–20 μm) were mounted on positively charged slides, dried and stored at −20 °C. We tried the following primary antibodies, all made in rabbit: Abgent AP18800C (1:250), Abcam ab81512 (1:250), Aviva ARP53315_P050 (1:125), and Biorbyt orb186523 (1:100), and used Alexa 488 goat anti-rabbit IgG secondary antibody (1:500) for all. We also tried Santa Cruz sc-103363 antibody, which was made in goat, with the Alexa 488 donkey anti-goat secondary IgG antibody.

### *Zpld1* mRNA *in situ* hybridization

Inner ears were dissected from neonatal mice after decapitation and fixed in 10% neutral buffered formalin (NBF) for 24 hours at room temperature. After two washes with 1xPBS (pH = 7.4), the samples were dehydrated in a series of ethanol solutions of increasing concentrations (70–100%), cleared by xylene immersion, and embedded in paraffin. 5 μm sections were cut using a microtome (Leica).

*In situ* hybridizations were performed using the RNAscope Assay^32^. The *Zpld1* probe was designed by Advanced Cell Diagnostics (ACDBio, USA) and consisted of a cocktail of short 20 bp oligonucleotides spanning the 160–1144 bp target region of the mouse *Zpld1* mRNA sequence (NM_178720.4). Hybridizations of the *Zpld1* probe and positive and negative control probes were performed with reagents and procedures described in the ACDBio User Manual for the RNAscope 2.5 HD Detection Kit (BROWN) using a Leica BOND autostainer.

### Whole-mount inner ear preparations

Inner ears from adult mice were dissected and flushed with neutral-buffered formalin (NBF) through a hole made at the cochlear apex. Inner ears were then immersed in NBF, dehydrated in ethanol, and cleared in methyl salicylate overnight. The preserved whole-mount preparations were examined for abnormalities under a dissecting microscope. To highlight the otolith organs, the cleared inner ears were visualized using polarized light because of its sensitivity to the refractive properties of otoconia.

### Auditory brainstem response (ABR) measurements

ABR thresholds of mice tested at the The Jackson Laboratory, presented in Fig. 1, were measured at 8, 16 and 32 kHz in a sound attenuating chamber using the SmartEP auditory evoked potential diagnostic system from Intelligent Hearing Systems (IHS, Miami, FL) as described previously^33^. Briefly, mice were anesthetized with tribromoethanol (0.2 ml of 20 mg/ml stock per 10 g of body weight, i.p.) and placed on a temperature controlled heating pad to maintain body temperature at 37° C. Output tubes from high frequency transducers were snugly fit in the ear canals of the mouse. Both ears were tested simultaneously, which effectively measures the thresholds of the better responding ear. Three subdermal electrodes, placed at the vertex and behind each ear, were used to record brain stem responses to defined tone-bursts (3 ms duration. 1.5 ms cosine-gated rise/fall time). The responses were then amplified, filtered (100–3000 Hz) and averaged (25 kHz sampling rate, 10 ms analysis window). Stimulus intensity was initially decreased in 10 dB steps until the response began to disappear and then lowered in 5 dB steps; ABR threshold was defined as the lowest intensity at which an ABR response could be reliably obtained. With our testing system, average ABR thresholds for normal hearing mice are about 40, 20, and 45 dB SPL for 8, 16 and 32 kHz stimuli, respectively.

ABR thresholds of animals tested at UNL, presented in Fig. 4, were measured according to previously described procedures^27,34,35^, which are similar to those given above with notable exceptions. Animals were anesthetized using a ketamine/xylazine mixture and prepared as described below for VsEP testing. Unilateral tone burst stimulation (1.0 ms rise-fall times with 1.0 ms plateau) at 8, 16 and 32 kHz was carried out using a high frequency transducer (SA1 driver, MF1 speaker, Tucker-Davis Technologies, Alachua, FL) coupled at the left ear via a modified commercial eartip (ER 10D-T03, Etymotic Research, Inc., Elk Grove Village, IL). Stimulus rate was 17 stimuli/s. Threshold was defined as the stimulus level midway between the minimum stimulus level producing a discernible response and the maximum level where no response was detectable. Stimuli were calibrated using a Bruel & Kjaar 1/8″ microphone and Nexus amplifier and levels expressed in terms of peak equivalent sound pressure level in dB peSPL.

### Vestibular sensory evoked potentials (VsEPs)

VsEPs were used to assess function of the inner ear otolithic organs. Animal preparation and functional testing with VsEPs followed procedures comparable to those previously described^35–39^. After arrival at UNL, animals were housed and maintained using standard husbandry methods until measurements were made.

During VsEP testing, mice were anesthetized with ketamine (90 to 126 mg/kg) and xylazine (10 to 14 mg/kg) injected intraperitoneally. Body core temperature was maintained at 37.0 ± 0.2 °C using a homeothermic heating blanket and rectal thermocouple (FHC, Inc., Bowdoin, ME). Subcutaneous stainless steel recording electrodes were placed over the skull at the nuchal crest (noninverting), behind the pinna (inverting) and at the hip (ground). Recorded electrical activity was amplified (200, 000x) and filtered (300 to 3000 Hz, −6 dB points) and led to a signal averager. Electrophysiological activity was digitized (1024 points, 10 microseconds/point) and used to produce response averages.

VsEPs were elicited by transient head translation stimuli (2 ms duration). The motion stimulus was a 2 ms linear jerk pulse, which was generated by a mechanical shaker (Labworks, Inc., Costa Mesa, CA; Model E2-203) driven by a power amplifier in response to a 2 ms linear voltage ramp control signal input. Linear jerk pulses (17 pulses/s) ranging in amplitude from +6 to −18 dB re: 1.0 g/ms (where 1.0 g = 9.8 m/s^2^) were adjusted in 3 dB steps and used to drive head translation in the naso-occipital axis. A broad band forward masker (50 to 50,000 Hz, 92 dB SPL) was presented during VsEP measurements^40^. A noninvasive head clip was used to secure the head to the mechanical shaker for delivery of the vestibular stimuli. Mice were placed in a supine position with nose up and stimuli were presented in the naso-occipital axis. Normal polarity began with upward head movement (naso-occipital +X). Inverted stimulus polarity began with downward movement (naso-occipital −X). Responses were collected for both normal and inverted stimulus polarities and the resulting 256 primary surface electrical response waveforms were averaged online to produce the final VsEP response for analysis.

The activity of the peripheral vestibular nerve has been shown to be reflected by the first positive (p1) and negative (n1) response peaks of the VsEP^41^. Therefore, amplitude and latency measures for the first response peaks (p1 and n1) of the waveform were quantified. Threshold was defined as the stimulus level midway between the minimum level that produced a discernible response and the maximum stimulus level that did not result in a visible response. Response peak latency was defined as the time, in microseconds (µs), from onset of the stimulus to the appearance of p1. Peak-to-peak amplitude, (p1-n1) measured in microvolts (µV), represented the difference between each positive peak and its respective negative peak. VsEP response threshold provided a measure of macular sensitivity to transient head motion whereas latencies reflected the activation timing and conduction of the vestibular compound action potential. VsEP amplitudes (p1-n1) provided an indicator of the number of synchronously firing vestibular neurons contributing to the VsEP response.

### Vestibulo-Ocular reflex (VOR) and optokinetic reflex (OKR) testing

VOR was used to assess semicircular canal and corresponding central reflex function, and the resulting metrics (gain and phase) provided the basis for quantitative analysis. OKR tests evaluated central oculo-motor circuits in the absence of head motion and thus examined to what extent, if any, these circuits could contribute to any VOR abnormalities. VOR methods performed at UNL are similar to those published by Stahl and colleagues^21,22,42–45^. VOR testing itself is performed non-invasively in the awake, unanesthetized animal.

Animals were prepared for VOR and OKR eye movement recordings by implanting a small neodymium magnet on the surface of the skull. All procedures were done under ketamine/xylazine anesthesia. A midline incision was made on the scalp extending from the bregma to the lambda, periosteum was removed to expose the parietal bones. The bone surface was prepared using a self-etch adhesive (Clearfil SE Bond 2, Kuraray Dental, New York, NY). An N42 neodymium magnet (3.2 × 0.3.2 × 1.6 mm, CMS Magnetics, Garland, TX) was attached to skull with light cured self-adhesive resin cement (RelyX Unicem 2, 3M Deutschland GmbH, Neuss, Germany). Post-operatively, an extended release preparation of non-steroidal anti-inflammatory drug (Meloxicam SR, Zoopharm, Windsor, CO) was administered for analgesia and prevention of inflammation. After surgery, animals were allowed to recover for at least 48 hours before eye movement recordings were made.

To limit pupil dilatation in the dark, the recording eye was pre-treated with 0.3–0.5% physostigmine salicylate ophthalmic solution 15 min before the start of recording. After briefly anesthetizing with Isoflurane, the mouse was restrained in a custom PVC tube animal holder by attaching the magnetic headpost to a magnetic bar that extended from the animal holder. The extended bar was oriented in such a way that the animal’s head was pitched ~30° nose down so that the horizontal semicircular canals were aligned with the horizontal plane^46,47^. The animal holder was secured to the center of a servo-controlled rotating table (Neuro Kinetics Inc, Pittsburgh, PA). VOR was evoked by rotating the animal in dark at 0.2 Hz, 0.4 Hz, 0.8 Hz, 1.6 Hz and 2.0 Hz with 25°/s peak velocity. Optokinetic stimulation used to elicit the OKR was provided by a black and white striped drum (stripe width 5°) that enclosed the animal and rotated in clockwise and anticlockwise directions at 10°/s peak velocity. Eye movements were recorded with a commercial pupil tracking system modified for rodents (ETL-200, ISCAN, Burlington, MA). Eye movement recordings and calibration procedures were similar to those previously described by^44^. Analog outputs of the ISCAN system and output from a rate sensor (Watson Pro Gyro 122-3A, Eau Claire, WI) attached to the platform on the rotating table were also recorded using a custom LabView routine for off-line analysis.

For data analysis, the positions of pupil and reference corneal reflection were converted to angular position as described previously^21,44^. Off-line analysis was performed using Origin Pro 2017 software (OriginLab, Northampton, MA). Segments of the recording with saccades and movement artifacts (eye blinks) were excluded from analysis. Three to five segments containing 6–10 cycles/segment were analyzed for each stimulus frequency. Eye position data was smoothed by frequency-constrained damped least squares curve fitting and differentiated to obtain the corresponding eye velocity. Gain and phase relationship were determined from sine-fits of table and eye velocities by four parameter sine-fits running up to 400 iterations of a Levenberg- Marquardt algorithm.

## Supplementary information

Supplementary Information

Supplementary Video-S1

Supplementary Video-S2

Supplementary Video-S3

## Data Availability

All data generated or analyzed during this study and access information for all shared biological materials are included in this published article (and its Supplementary Information Files).
