# Supplementary material for: Spontaneous mutations of the Zpld1 gene in mice cause semicircular canal dysfunction but do not impair gravity receptor or hearing functions
Source: Sci Rep. 2019 Aug 27;9:12430. doi: 10.1038/s41598-019-48835-5 (PMC6711997; doi:10.1038/s41598-019-48835-5)

Electronic supplementary material

Spontaneous mutations of the *Zpld1* gene in mice cause semicircular canal dysfunction but do not impair gravity receptor or hearing functions

Sarath Vijayakumar[1], Sherri M. Jones[1], Timothy A. Jones[1], Cong Tian[2], Kenneth R. Johnson[2]

1. Department of Special Education and Communication Disorders, University of Nebraska, Licoln, NE, USA
2. The Jackson Laboratory, Bar Harbor, ME, USA

Contents

Supplementary Video S1. Behavior of a *cwh/cwh* mouse compared with control.

A 12-week-old *Zpld1*$^{cwh/cwh}$ homozygous mutant mouse exhibits hyperactivity and circling behavior compared with an age-matched non-mutant control.

Supplementary Video S2. Behavior of a *sprl/sprl* mouse compared with control.

A 9-week-old *Zpld1*$^{sprl/sprl}$ homozygous mutant mouse exhibits hyperactivity and circling behavior compared with an age-matched non-mutant control mouse.

Supplementary Video S3. Behavior of a *sprl/-* mouse compared with control.

A 6-week-old *Zpld1*$^{sprl/-}$ compound heterozygous mutant mouse exhibits hyperactivity and circling behavior compared with an age-matched non-mutant control mouse.

Figure S1. All mutant mice have normal hearing thresholds. ABR threshold means and their associated standard errors are shown for +/*sprl* (5f, 2m), *sprl/sprl* (8 f) +/*cwh* (6f, 3m), *cwh/cwh* (4f, 4m), and +/+ (6f, 10 m) mice at 4-8 wks of age. There are no statistically significant differences between ABR threshold means for any of the test frequencies.

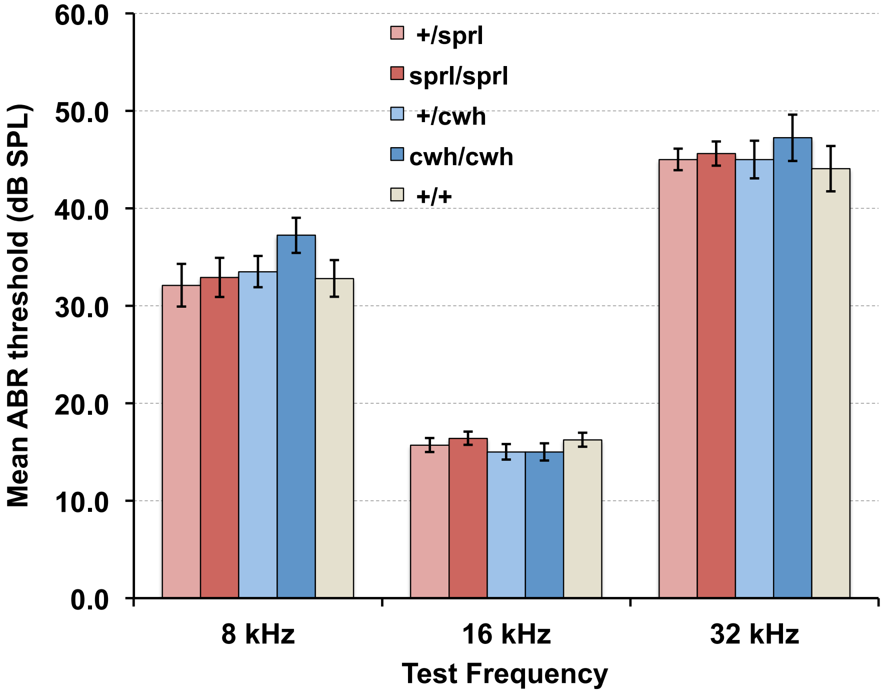

Figure S2. Cleared, whole mounts of the inner ears from a 70-day-old *cwh/cwh* mouse viewed under polarizing light. No morphological abnormalities of the inner ears are apparent, and the otoconia in both the utricle (u) and saccule (s) appear normal. Compare with Figure 1B.

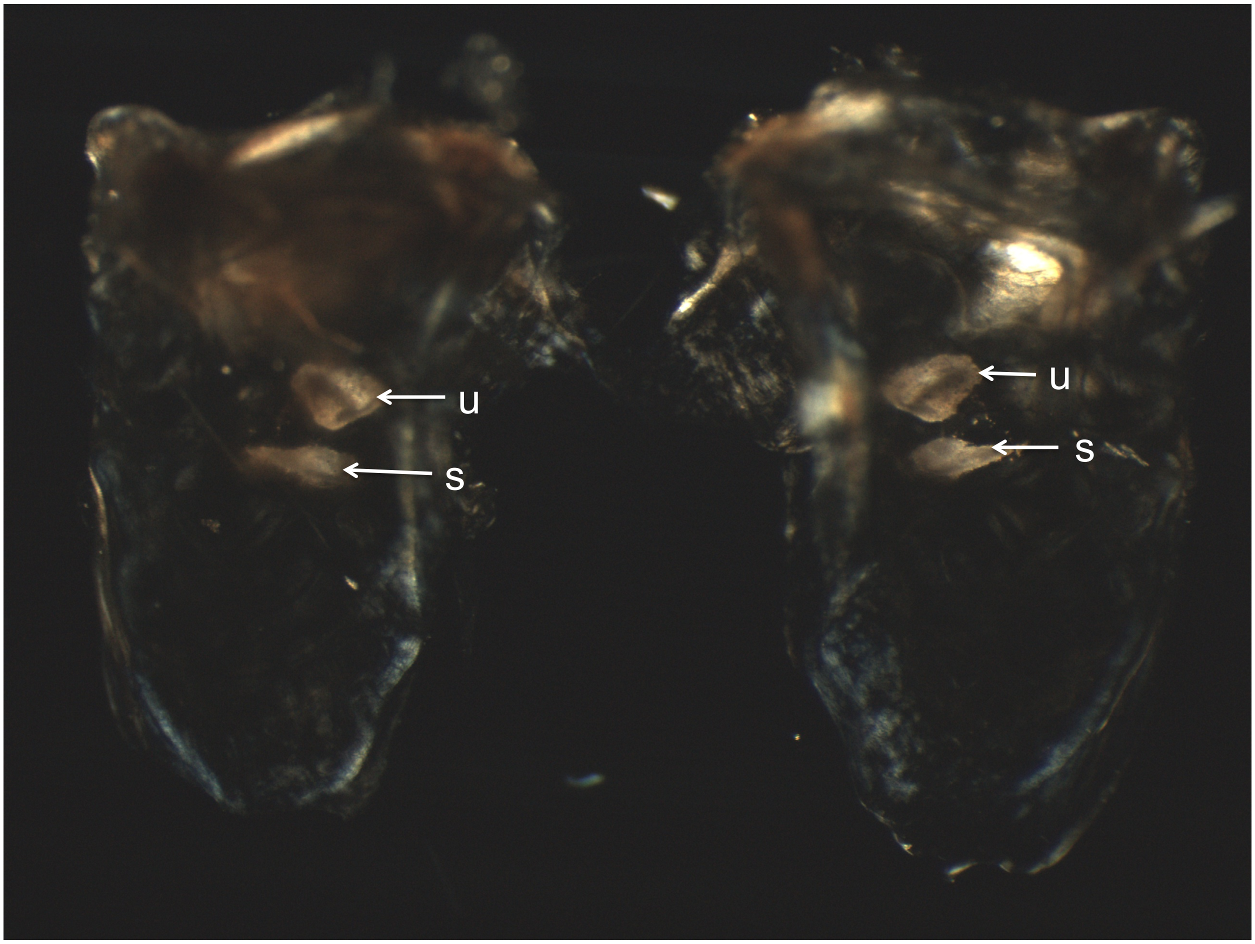

Supplement: Supplementary file 1 — Supplementary Information [file 41598_2019_48835_MOESM1_ESM.pdf]
